# Comparison of Real-Time Fluorescence Confocal Digital Microscopy With Hematoxylin-Eosin–Stained Sections of Core-Needle Biopsy Specimens

**DOI:** 10.1001/jamanetworkopen.2020.0476

**Published:** 2020-03-05

**Authors:** Savitri Krishnamurthy, Sharjeel Sabir, Kechen Ban, Yun Wu, Rahul Sheth, Alda Tam, Funda Meric-Bernstam, Kenna Shaw, Gordon Mills, Roland Bassett, Stanley Hamilton, Marshall Hicks, Sanjay Gupta

**Affiliations:** 1 Division of Pathology and Laboratory Medicine, The University of Texas MD Anderson Cancer Center, Houston; 2 Department of Radiology, Scripps Mercy Hospital, San Diego, California; 3 Department of Interventional Radiology, The University of Texas MD Anderson Cancer Center, Houston; 4 Department of Investigational Cancer Therapeutics, The University of Texas MD Anderson Cancer Center, Houston; 5 Sheikh Khalifa Bin Zayed Al Nahyan Institute for Personalized Cancer Therapy, The University of Texas MD Anderson Cancer Center, Houston; 6 Oregon Health and Science University Knight Cancer Institute, Portland; 7 Department of Biostatistics, The University of Texas MD Anderson Cancer Center, Houston

## Abstract

**Importance:**

Strategies to procure high-quality core-needle biopsy (CNB) specimens are critical for making basic tissue diagnoses and for ancillary testing.

**Objectives:**

To investigate acquisition of fluorescence confocal microscopy (FCM) images of interventional radiology (IR)–guided CNB in real time in the radiology suite and to compare the accuracy of FCM diagnoses with those of hematoxylin-eosin (H&E)–stained CNB sections.

**Design, Setting, and Participants:**

In this diagnostic study, FCM imaging of IR-guided CNBs was performed in the radiology suite at a major cancer center for patients with an imaging abnormality from August 1, 2016, to April 30, 2019. The time taken to acquire FCM images and the quality of FCM images based on percentage of interpretable tissue with optimal resolution was recorded. The FCM images were read by 2 pathologists and categorized as nondiagnostic, benign/atypical, or suspicious/malignant; these diagnoses were compared with those made using H&E-stained tissue sections. Cases with discrepant diagnosis were reassessed by the pathologists together for a consensus diagnosis. Data were analyzed from June 3 to July 19, 2019.

**Interventions:**

Each IR-guided CNB was stained with 0.6mM acridine orange, subjected to FCM imaging, and then processed to generate H&E-stained sections.

**Main Outcomes and Measures:**

Mean time taken for acquisition of FCM images, quality of FCM images based on interpretable percentage of the image, and accuracy of diagnostic categorization based on FCM images compared with H&E-stained sections.

**Results:**

A total of 105 patients (57 male [54.3%]; mean [SD] age, 63 [13] years) underwent IR-guided CNBs in a mean (SD) of 7 (2) minutes each. The FCM images showed at least 20% of the tissue with optimal quality in 101 CNB specimens (96.2%). The FCM images were accurately interpreted by the 2 pathologists in 100 of 105 cases (95.2%) (2 false-positive and 3 false-negative) and 90 of 105 cases (85.7%) (6 false-positive and 9 false-negative). A reassessment of 14 discordant diagnoses resulted in consensus diagnoses that were accurate in 101 of 105 cases (96.2%) (1 false-positive and 3 false-negative).

**Conclusions and Relevance:**

The ease of acquisition of FCM images of acceptable quality and the high accuracy of the diagnoses suggest that FCM may be useful for rapid evaluation of IR-guided CNBs. This approach warrants further investigation.

## Key Points

**Question:**

Can fluorescence confocal microscopy be used for immediate evaluation of interventional radiology–guided core-needle biopsy in real time at the bedside in the radiology suite?

**Findings:**

In this diagnostic study of 105 patients undergoing core-needle biopsy, fluorescence confocal microscopy images were acquired in a mean of 7 minutes, were similar to hematoxylin-eosin–stained tissue sections, and were of acceptable quality in 101 (96.2%). The accuracy of fluorescence confocal microscopy diagnosis compared with the criterion standard of hematoxylin-eosin–stained tissue sections was 95.2% for pathologist 1, 85.7% for pathologist 2, and 96.2% for consensus between them.

**Meaning:**

These findings suggest that fluorescence confocal microscopy can be used for real-time bedside evaluation of interventional radiology–guided core-needle biopsy with high accuracy in a range of medical settings for procuring high-quality specimens in 1 hospital visit.

## Teaser

This diagnostic study investigates real-time acquisition of fluorescence confocal microscopy of interventional radiology–guided specimens in the radiology suite and compares accuracy of diagnoses with hematoxylin-eosin (H&E)–stained sections among patients undergoing core-needle biopsy.

## Introduction

Interventional radiology (IR)–guided core-needle biopsy (CNB) is the most commonly used procedure for procuring tissue for the investigation of radiologically identified abnormalities in solid organs. A high-quality CNB specimen with adequate cellularity that is representative of the lesion lays the foundation for success with basic diagnostic pathology reporting and for completion of the validated ancillary genomic and immunohistochemical testing that may be required in selected patients for personalized treatment. Our ability to realize the potential of personalized medicine depends on the availability of CNB specimens with optimal tumor cellularity together with preserved tissue integrity.^1^ The lack of high-quality tissue specimens is a critical roadblock to developing and validating cancer biomarkers.^2^ A large number of clinical trials fail to perform pharmacodynamic analyses or report incomplete findings from such analyses owing to suboptimal tissue biopsies.^3,4^ At present, as many as 15% of CNB specimens procured from solid tumors demonstrate insufficient tissue for ancillary molecular testing using any of the currently available genomic platforms.^5^ There is ongoing discussion to improve the acquisition of high-quality CNB specimens for patient care and for successful completion of ancillary testing in clinical trials.^6,7,8^ A minimum tumor cellularity of 20% is generally required for ancillary genomic testing using next-generation sequencing.^9^ Strategies to ensure high-quality CNBs are important to avoid second visits to the hospital for the sole purpose of procuring tissue with optimal tumor cellularity.

The current technique for rapid evaluation of CNB specimens is touch preparation, which can demonstrate high levels of sensitivity and specificity in indicating representative sampling of the malignant tumor but cannot accurately estimate tumor cellularity in the specimen.^10,11^ In addition, touch preparation can distort the tissue with suboptimal preservation for subsequent histopathologic examination.^12,13^ Frozen section analysis is not used for rapid assessment of CNB specimens because of loss of tissue in the cryostat when the frozen sections are cut. Better modalities to evaluate the quality of specimens at the bedside in real time are needed to ensure procurement of high-quality CNB specimens and avoid a second visit for repeating the biopsy.

A commercially available fluorescence confocal microscopy (FCM) platform (Caliber ID, Inc) has been shown to be feasible for ex vivo imaging of small fragments of residual tissue from surgical resections of different organs.^14,15^ We conducted a prospective study in the radiology suite to evaluate the use of FCM for real-time bedside ex vivo tissue imaging of IR-guided CNBs. The primary objectives of our study were to evaluate the ability to acquire FCM images of CNB tissue at the bedside in the radiology suite and the accuracy of the diagnoses made using these FCM images compared with the criterion standard of histopathologic examination of hematoxylin-eosin (H&E)–stained tissue sections of the CNB tissue.

## Methods

This diagnostic study was conducted at The University of Texas MD Anderson Cancer Center, Houston, under an institutional review board–approved protocol with written informed consent to image 1 IR-guided CNB from each participant from August 1, 2016, to April 30, 2019. The study followed the Standards for Reporting of Diagnostic Accuracy (STARD) reporting guideline.

The IR physicians (S.S., R.S., A.T., M.H., and S.G.) evaluated the safety of IR-guided biopsy and determined the best imaging modality for biopsy guidance (ultrasonography or computed tomography) in patients with suspected solid tumors. Biopsy procedures were performed using the coaxial technique, and CNB specimens were obtained using a semiautomatic side-cutting core gun set (Quick-Core [Cook Medical] or Mission [Bard]) that allowed the procurement of a 1- or 2-cm-long CNB specimen.

We received 1 CNB specimen per patient from the radiologist (S.S., R.S., A.T., M.H., and S.G.) in the IR suite; the specimen was placed in a petri dish, stained with 0.6mM acridine orange for approximately 10 seconds, and then imaged in the radiology suite using a FCM platform (Vivascope 2500 RSG4; Caliber ID, Inc). The CNB specimen was scanned using light at 488 and 785 nm to obtain grayscale and digitally colorized FCM images, respectively. The time taken to image the specimens was recorded from receipt of the specimen to acquisition of the FCM digital images. The FCM platform was designed specifically for ex vivo imaging of fresh biological tissue specimens and includes a diode laser that operates at wavelengths of 488 and 785 nm, a 550-nm bandpass filter, a maximum illumination power of 5 mW, and an oil immersion objective lens with a numerical aperture of 0.9 and original magnification ×40. At these settings, the lateral resolution was 1.0 μm, and the axial resolution was less than 5.0 μm. Images were acquired at 9 frames per second, and the composite image was 2.0 cm at the greatest diameter. After completion of FCM imaging, the CNB tissue was fixed in 10% formalin, processed routinely to generate 5-μm formalin-fixed, paraffin-embedded tissue blocks, and stained using H&E for histopathologic examination.

The FCM grayscale and digitally pseudocolorized blue images for each specimen were examined in detail at various original magnifications corresponding to ×1 to ×60 of a conventional light microscope at the site of acquisition or remotely using TeamViewer software, version 14.2.8352 (TeamViewer) at the end of the study. The FCM images were interpreted by 2 board-certified pathologists (S.K. and Y.W.) who were blinded to the final histopathologic interpretation of the H&E-stained sections. Pathologist 1 (S.K.) had more experience interpreting FCM images, whereas pathologist 2 (Y.W.) was trained using a study set of FCM images. The quality of the images was graded semiquantitatively according to the extent of tissue architecture recognized and quality of the image, where 0 indicates none of the image was interpretable; 1, less than 20% was interpretable; 2, at least 20% to less than 50% was interpretable; and 3, at least 50% was interpretable. The FCM images were categorized by both study pathologists as nondiagnostic, benign, atypical, suspicious, or malignant, and for cases categorized as suspicious or malignant, a specific histologic diagnosis was made and the percentage of tumor cellularity was determined. The diagnoses made using FCM were compared with diagnoses made using H&E-stained sections with regard to diagnostic categorization, estimation of tumor cellularity, and specific diagnoses. The H&E-stained sections were interpreted by pathologist 1, who was blinded to the results of FCM interpretation. The H&E results from pathologist 1 were compared with those from the pathologist who interpreted the CNB results to generate the final pathology report for clinical care of the patient. Both study pathologists together reassessed cases with discordant FCM diagnoses to arrive at a consensus diagnosis.

### Statistical Analysis

We determined the mean time for acquisition of each FCM image; the quality of the FCM images; and the sensitivity, specificity, accuracy, and positive and negative predictive value of diagnostic categorization of FCM images in comparison with the criterion standard of H&E-stained sections of the imaged CNB. We used the Cohen κ statistic to summarize interreader agreement for diagnostic categorization of FCM images. Pearson correlation analysis was used to compare the estimations of tumor cellularity between the FCM interpretations of the 2 study pathologists and between the interpretations made by each pathologist based on the FCM images and the H&E-stained sections. All statistical analyses were performed using R, version 3.6.0 (R Project for Statistical Computing).

## Results

We recruited 105 patients (57 male [54.3%] and 48 female [45.7%]; mean [SD] age, 63 [13] years) for FCM imaging of IR-guided CNBs from a variety of sites, including lung, liver, adrenal gland, kidney, bone, pleura, lymph node, and soft tissue, in this diagnostic study. Table 1 shows the sources of the CNBs, mean size of the lesions, types of radiology guidance used, and needle gauges. The time from receipt of the specimen from the interventional radiologist to acquisition of FCM images ranged from 3 to 13 minutes (mean [SD], 7 [2] minutes). The quality of the FCM images was scored as 3 by both pathologists in 59 patients (56.2%), as 2 or 3 in 42 patients (40.0%), and as 1 in 4 patients (3.8%).

**Table 1.** Specimen Sources, Radiology Guidance, Lesion Sizes, and Needle Gauges Used for Procuring Core-Needle Biopsy Specimens

Source of Specimen	No. (%) of Cases^a^						Size, Mean (SD), cm
	All	Type of Radiology Guidance		Core-Needle Size			
		CT	US	20 Gauge	18 Gauge	14 Gauge	
Adrenal gland	5 (4.8)	5 (6.0)	0	0	5 (5.7)	0	3.2 (1.5)
Bone	8 (7.6)	8 (9.6)	0	0	5 (5.7)	3 (100)	4.0 (1.7)
Kidney	8 (7.6)	7 (8.4)	1 (4.5)	2 (13.3)	6 (6.9)	0	4.4 (2.9)
Liver	13 (12.4)	4 (4.8)	9 (40.9)	0	13 (14.9)	0	3.4 (2.9)
Lung	15 (14.3)	15 (18.1)	0	13 (86.7)	2 (2.3)	0	3.5 (2.3)
Lymph node	15 (14.3)	12 (14.5)	3 (13.6)	0	15 (17.2)	0	1.9 (0.9)
Pleura	2 (1.9)	1 (1.2)	1 (4.5)	0	2 (2.3)	0	3.0 (1.4)
Soft tissue	39 (37.1)	31 (37.3)	8 (36.4)	0	39 (44.8)	0	3.5 (2.3)
All	105 (100)	83 (100)	22 (100)	15 (100)	87 (100.0)	3 (100)	3.3 (2.4)

Abbreviations: CT, computed tomography; US, ultrasonography.

^a^ Percentages have been rounded and may not total 100.

The H&E-stained tissue sections of the CNB specimen demonstrated similar quality as the nonimaged sections and were interpreted by pathologist 1 as nondiagnostic in 5 patients (4.8%), as benign or atypical in 24 (22.9%), and as suspicious or malignant in 76 (72.4%). We found no discrepancy in the categorization of the H&E-stained sections between pathologist 1 and the histologic diagnosis in the final pathology report.

The nuclear staining of the tissue with acridine orange created the necessary contrast between the nucleus and cytoplasm to allow the recognition of tissue architecture in the grayscale and digitally pseudocolorized blue FCM images. Pathologist 1 interpreted the FCM images as nondiagnostic in 5 patients (4.8%), benign or atypical in 25 (23.8%), and suspicious or malignant in 75 (71.4%). Pathologist 2 interpreted the FCM images as nondiagnostic in 5 patients (4.8%), benign or atypical in 27 (25.7%), and suspicious or malignant in 73 (69.5%). Pathologist 1 correctly diagnosed 100 patients (95.2%), and pathologist 2 correctly diagnosed 90 (85.7%) (Table 2). Pathologist 1 made 2 false-positive diagnoses (1 each from soft tissue and lymph node) and 3 false-negative diagnoses (2 from bone and 1 from soft tissue). Pathologist 2 made 6 false-positive diagnoses (2 from liver, 2 from soft tissue, and 1 each from bone and lung) and 9 false-negative diagnoses (3 from bone, 3 from lymph node, and 1 each from soft tissue, kidney, and liver). The quality of the FCM images in the misdiagnosed cases was 2 in 4 cases and 3 in the 16 remaining cases. The false-positive diagnoses resulted from misdiagnosis of reactive myofibroblastic proliferation or inflammation with many histiocytes as malignant tumor, and the false-negative diagnoses resulted from lack of recognition of malignant tumor of very low cellularity, ranging from 2% to 5%, in the CNB specimen. The 2 pathologists reassessed the 14 discrepant cases, which resulted in 1 false-positive diagnosis (from soft tissue), in which reactive myofibroblastic proliferation was misinterpreted as sarcoma, and 3 false-negative diagnoses of small foci of metastatic carcinoma (2 cases from bone and 1 from lymph node). The remaining 10 cases received accurate consensus diagnoses, for a final accurate diagnosis in 101 of 105 cases (96.2%).

**Table 2.** Categorization of the Fluorescence Confocal Microscopy Images by the 2 Pathologists

Category	No. (%) of CNB Specimens^a^			
	Pathologist		Diagnosis	
	1	2	Consensus	H&E Stain
Nondiagnostic	5 (4.8)	5 (4.8)	5 (4.8)	5 (4.8)
Benign or atypical	25 (23.8)	28 (26.7)	26 (24.8)	24 (22.9)
Suspicious or malignant	75 (71.4)	72 (68.6)	74 (70.5)	76 (72.4)
Total	105 (100)	105 (100)	105 (100)	105 (100)

Abbreviations: CNB, core-needle biopsy; H&E, hematoxylin-eosin.

^a^ Percentages have been rounded and may not total 100.

**Figure 1.**
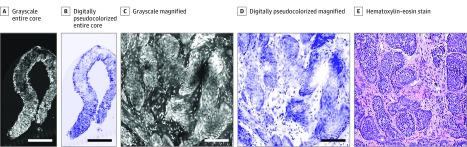
Images of Core-Needle Biopsy Specimen Obtained From Bone Grayscale and digitally pseudocolorized fluorescence confocal microscopy images of interventional radiology–guided core-needle biopsy specimens and corresponding hematoxylin-eosin–stained tissue section from bone that were accurately diagnosed by both study pathologists independently using fluorescence confocal microscopy. The fluorescence confocal microscopy images resemble the hematoxylin-eosin–stained images and demonstrate the presence of poorly differentiated carcinoma. Scale bar indicates 1500 μm in A and B and 100 μm in C and D. Original magnification ×100 in E.

**Figure 2.**
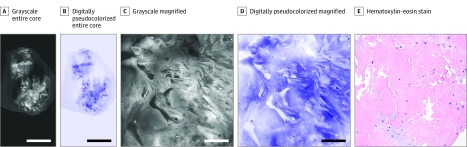
Images of Core-Needle Biopsy Specimen Obtained From Lung Grayscale and digitally pseudocolorized fluorescence confocal microscopy images and corresponding hematoxylin-eosin–stained tissue section of interventional radiology–guided core-needle biopsy from lung. The core-needle biopsy is nondiagnostic, including fibroconnective tissue alone, which is well recognized in the fluorescence confocal microscopy images. Scale bar indicates 1500 μm in A and B and 100 μm in C and D. Original magnification ×100 in E.

The specific diagnoses made by the study pathologists independently using the FCM images correlated perfectly with the H&E diagnoses. Figure 1 shows FCM images with the corresponding image of H&E-stained tissue section of CNB obtained from bone that was correctly recognized by both pathologists. Figure 2 shows FCM and H&E-stained images of CNB from lung that was nondiagnostic, including fibroconnective tissue alone, which was accurately interpreted on FCM images by the study pathologists. Additional FCM and corresponding H&E-stained images of 6 optimal and 3 nondiagnostic and suboptimal cases of CNBs obtained from different sites that were correctly recognized by both pathologists are shown as eFigure 1 and eFigure 2 in the Supplement.

In cases diagnosed as malignant, the correlation of the estimated tumor cellularity on the FCM images with that of the corresponding H&E-stained sections was 0.584 for pathologist 1 and 0.424 for pathologist 2. A total of 29 cases (27.6%), including 5 nondiagnostic cases and 24 cases with less than 20% tumor cellularity, were identified on H&E-stained sections as not suitable for ancillary genomic testing. Of these cases, 20 (69.0%) were deemed suboptimal on FCM interpretation and estimation of tumor cellularity; in the remaining cases, the estimation of tumor cellularity was above the 20% cutoff, with 20% to 30% estimated tumor cellularity on FCM images.

The κ statistic for interreader agreement for the categorization of the FCM images was 0.679 (95% CI, 0.526-0.831). The correlation of the estimations of tumor cellularity between the 2 pathologists was 0.732. The accuracy level of interpretation of the FCM images compared with the criterion standard of H&E diagnosis was 95.2% for pathologist 1, 85.7% for pathologist 2, and 96.2% for the consensus between pathologists 1 and 2. Table 3 shows the overall performance of both pathologists independently and after reassessment of the discrepant cases, including sensitivity, specificity, accuracy, positive predictive value, and negative predictive value in the interpretation of the FCM images.

**Table 3.** Independent Performance

Reader	Performance, %^a^				
	Sensitivity	Specificity	Accuracy	PPV	NPV
Pathologist 1	96.0	91.6	95.2	97.3	88.0
Pathologist 2	86.8	79.1	85.7	92.9	65.5
Consensus	97.3	91.6	96.2	97.3	91.6

Abbreviations: NPV, negative predictive value; PPV, positive predictive value.

^a^ Performance by both pathologists was measured after consensus diagnosis of discrepant cases in the interpretation of fluorescent confocal microscopy images in comparison with the criterion standard of hematoxylin-eosin–stained sections.

## Discussion

Procuring high-quality CNB specimens that are representative of the area of imaging abnormality and that contain adequate tissue cellularity is a top priority in any medical practice. Our results show the promising utility of FCM for real-time imaging of IR-guided CNBs at the bedside in the radiology suite. The ease of acquisition of digital images of acceptable quality within 7 minutes, together with the high accuracy of diagnosis, demonstrates the suitability of FCM for real-time bedside tissue evaluation in clinical practice. The FCM images resembled light microscopy of H&E-stained sections, could be interpreted onsite or remotely, and enabled diagnostic categorization of the CNB specimen. The lack of uniform penetration of light due to variable tissue thickness resulted in focal areas with less-than-optimal resolution. However, the problem of small areas in the FCM image that appeared bleached and less sharp did not compromise recognition of tissue architecture to arrive at a definite diagnosis. In addition, the CNB specimen was preserved optimally for subsequent tissue processing, as evidenced by the quality of the H&E-stained sections. The less-than-optimal correlation of the estimation of tumor cellularity between FCM images and H&E-stained sections was most likely related to alterations in tumor cellularity when deeper cuts into the tissues were made after processing the imaged CNB tissue. Furthermore, all the nondiagnostic (5 of 105) CNBs and those with less than 20% tumor cellularity (24 of 105) on H&E-stained sections that would be regarded as suboptimal for ancillary genomic testing were correctly recognized on FCM images in the study.

The false-negative FCM diagnoses included CNB specimens with less than 20% tumor cellularity that would be rated as suboptimal for ancillary studies. The false-positive diagnoses resulted from misinterpretation of reactive myofibroblastic proliferation or inflammation, fibrosis, and infiltration of histiocytes as malignant epithelial tumor. The marginally better performance of pathologist 1 was most likely owing to more experience in interpreting FCM images. The accuracy of FCM diagnosis was lowered purely owing to interpretive errors and not because of the quality, source, tumor type, or size of the CNB specimen. No particular organ of origin of the FCM images was difficult or easy to interpret by either pathologist in the study.

Optical imaging modalities are emerging as the next generation of digital microscopy tools, with implications for pathology practice.^16,17^ We chose the commercially available investigational prototype of an FCM platform for real-time bedside imaging of IR-guided CNBs. This FCM device has been extensively tested for evaluating skin specimens obtained from Mohs surgery for diagnosis and margin assessment of basal cell carcinoma.^18,19,20^ The feasibility of using FCM for ex vivo tissue imaging of nonskin specimens has also been demonstrated recently.^21,22,23,24^ A previous study using an earlier version of the FCM device to image 23 ultrasonography-guided CNB specimens obtained from patients with inflammatory breast cancer^25^ showed moderate agreement between invasive tumor cellularity estimated using grayscale FCM images and that estimated using H&E-stained sections. Very recently, Puliatti et al^26^ used the same imaging device that we used in the present study to evaluate 18-gauge punch biopsy specimens from the prostate that were obtained from 13 patients who underwent robot-assisted laparoscopic radical prostatectomy. Three pathologists interpreted 89 punch biopsy specimens; 91% of the specimens were diagnosed correctly, and sensitivity and specificity were 83% and 91%, respectively. However, our study is the first, to our knowledge, to show the utility of FCM for real-time bedside imaging of IR-guided CNB specimens from a variety of sites belonging to different tumor types in the radiology suite. An FCM device (Caliber ID, Inc) similar to that used in our study is designated by the US Food and Drug Administration as a class I microscopy device and is available commercially for imaging clinical specimens. Other optical techniques, such as laser scanning microscopy, microscopy with UV surface excitation, and structured illumination microscopy, can also be used for imaging CNB specimens and can generate digitally colorized images to resemble H&E-stained sections, similar to those generated by FCM.^27,28,29,30^ However, the utility of any of these techniques has not been tested beyond the initial feasibility studies, and none of these modalities are yet available commercially. Preservation of tissue integrity of acridine orange–stained and imaged tissue for conventional histopathologic examination has been demonstrated by other investigators, with findings similar to ours.^31,32,33^ In addition, optimal preservation of acridine orange–stained and imaged tissue for subsequent immunohistochemistry and genomic studies has been established recently.^32,34^ The imaged CNBs can potentially be used for immunohistochemical analysis and genomic testing after formalin fixation and routine processing. The functional requirements for an ideal ex vivo microscopy device suitable for margin assessment of surgical resections, evaluation of tissue adequacy for diagnosis, and identification of the area of lesion in the tissue for special studies or for biorepository purposes were recently described in a study sponsored by the College of American Pathologists^35^; these desired features include size and portability of the device, specimen preparation, imaging time, field of view, resolution, diagnostic capability, yield, accuracy, ease of use, and safety. The FCM device used in our study fulfills these requirements and thus may be suitable for routine surgical pathology practice.

### Limitations

The study had some limitations. Although the FCM images of IR-guided CNB specimens were acquired in real time at the bedside in the radiology suite, they were not interpreted immediately. Therefore, the radiologists performing the biopsy did not receive immediate feedback regarding the quality of the CNB. The tissue integrity of the imaged IR-guided CNB specimen was proven for conventional H&E staining alone and not for ancillary immunohistochemical or genomic testing. Our results need further validation in a multi-institutional study including a larger sample size and interpretation of FCM images by many pathologists practicing in an academic as well as community settings.

## Conclusions

Our FCM study of IR-guided CNB demonstrates the utility of this next-generation digital microscopy technique for obtaining grayscale and digitally pseudocolorized images of IR-guided CNB specimens in real time in the radiology suite. The inherently digital FCM images resembling images of H&E-stained sections can be interpreted at the bedside or remotely by pathologists. This modality can be easily embraced by pathology practices to facilitate the acquisition and rapid evaluation of high-quality, diagnostic CNB specimens in 1 visit to the hospital. Real-time bedside evaluation of CNB specimens can improve the acquisition of high-quality biospecimens not only for effective clinical practice but also for successful correlative studies in clinical trials. The potential benefits of immediately triaging the imaged CNB for ancillary molecular testing need further investigation.

## Notes

Supplement. **eFigure 1.** Specimens From Liver, Lung, Lymph Node, Pleura, Soft Tissue, and Kidney **eFigure 2.** Specimens From Soft Tissue and Liver
