# Supplementary material for: Comparison of Real-Time Fluorescence Confocal Digital Microscopy With Hematoxylin-Eosin–Stained Sections of Core-Needle Biopsy Specimens
Source: JAMA Netw Open. 2020 Mar 5;3(3):e200476. doi: 10.1001/jamanetworkopen.2020.0476 (PMC7059022; doi:10.1001/jamanetworkopen.2020.0476)
Supplement: Supplement. — eFigure 1. Specimens From Liver, Lung, Lymph Node, Pleura, Soft Tissue, and Kidney eFigure 2. Specimens From Soft Tissue and Liver [file jamanetwopen-3-e200476-s001.pdf]

Supplementary Online Content

Krishnamurthy S, Sabir S, Ban K, et al. Comparison of real-time fluorescence confocal digital microscopy with hematoxylin-eosin–stained sections of core-needle biopsy specimens. *JAMA Netw Open*. 2020;3(3):e200476. doi:10.1001/jamanetworkopen.2020.0476

eFigure 1. Specimens From Liver, Lung, Lymph Node, Pleura, Soft Tissue, and Kidney

eFigure 2. Specimens From Soft Tissue and Liver

This supplementary material has been provided by the authors to give readers additional information about their work.

©2020 Krishnamurthy S et al. *JAMA Network Open*.

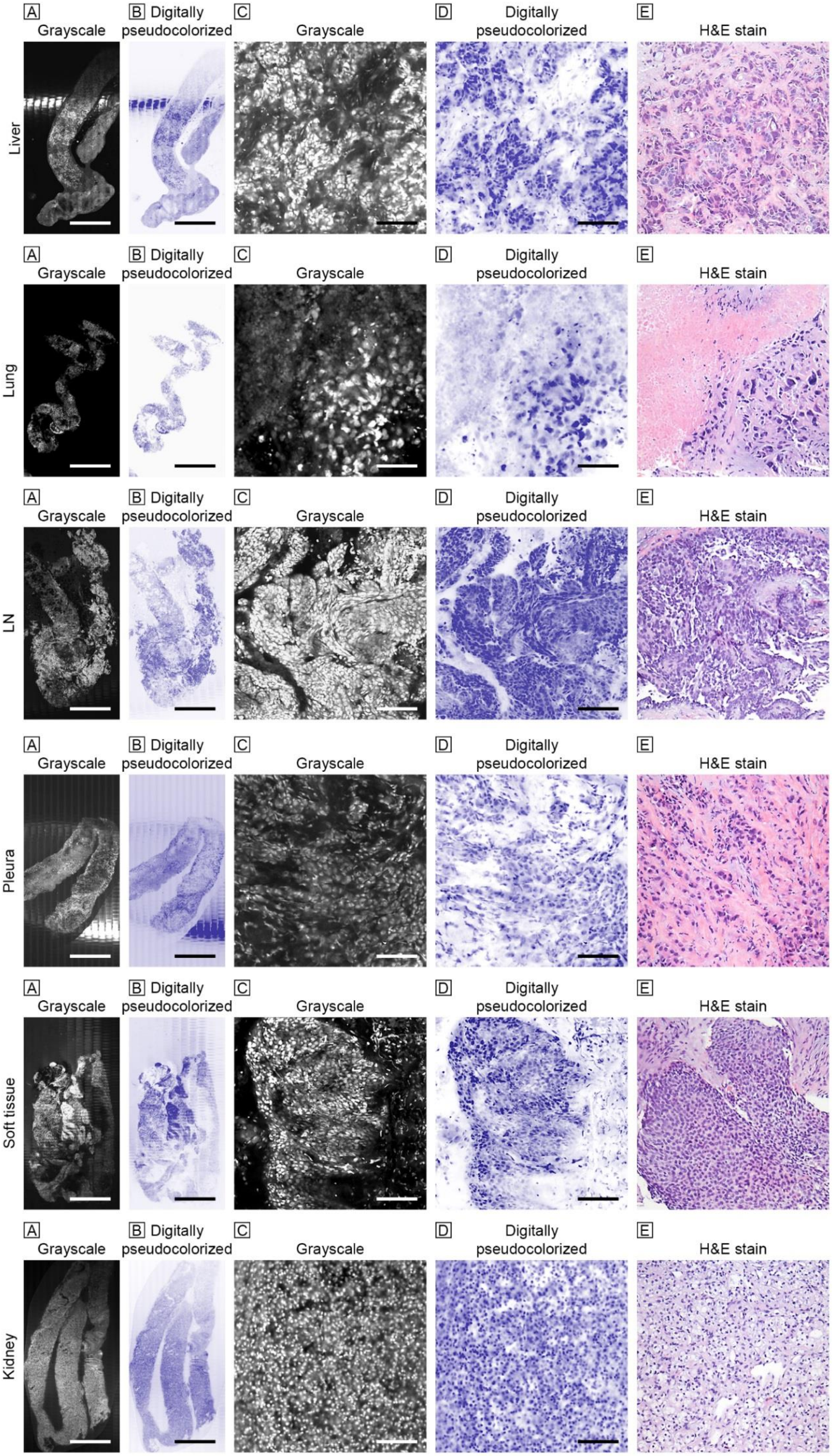

©2020 Krishnamurthy S et al. *JAMA Network Open*.

eFigure 1. Specimens From Liver, Lung, Lymph Node, Pleura, Soft Tissue, and Kidney

Grayscale (entire core in A, higher magnification in C) and digitally pseudocolorized (entire core in B,higher magnification in D) fluorescence confocal microscopy (FCM) images of interventional radiology–guided core needle biopsies (CNB) and corresponding hematoxylin and eosin (H&E)–stained tissue sections (E) from liver, lung, lymph node, pleura, soft tissue, and kidney biopsies that were accurately diagnosed by the study pathologists independently using FCM. The FCM images resemble the H&E images and demonstrate the presence of poorly differentiated carcinoma. (Magnification: 1500 µM scale bar in columns A and B. 100 µM scale bar in columns C and D. X100 in column E).

©2020 Krishnamurthy S et al. *JAMA Network Open*.

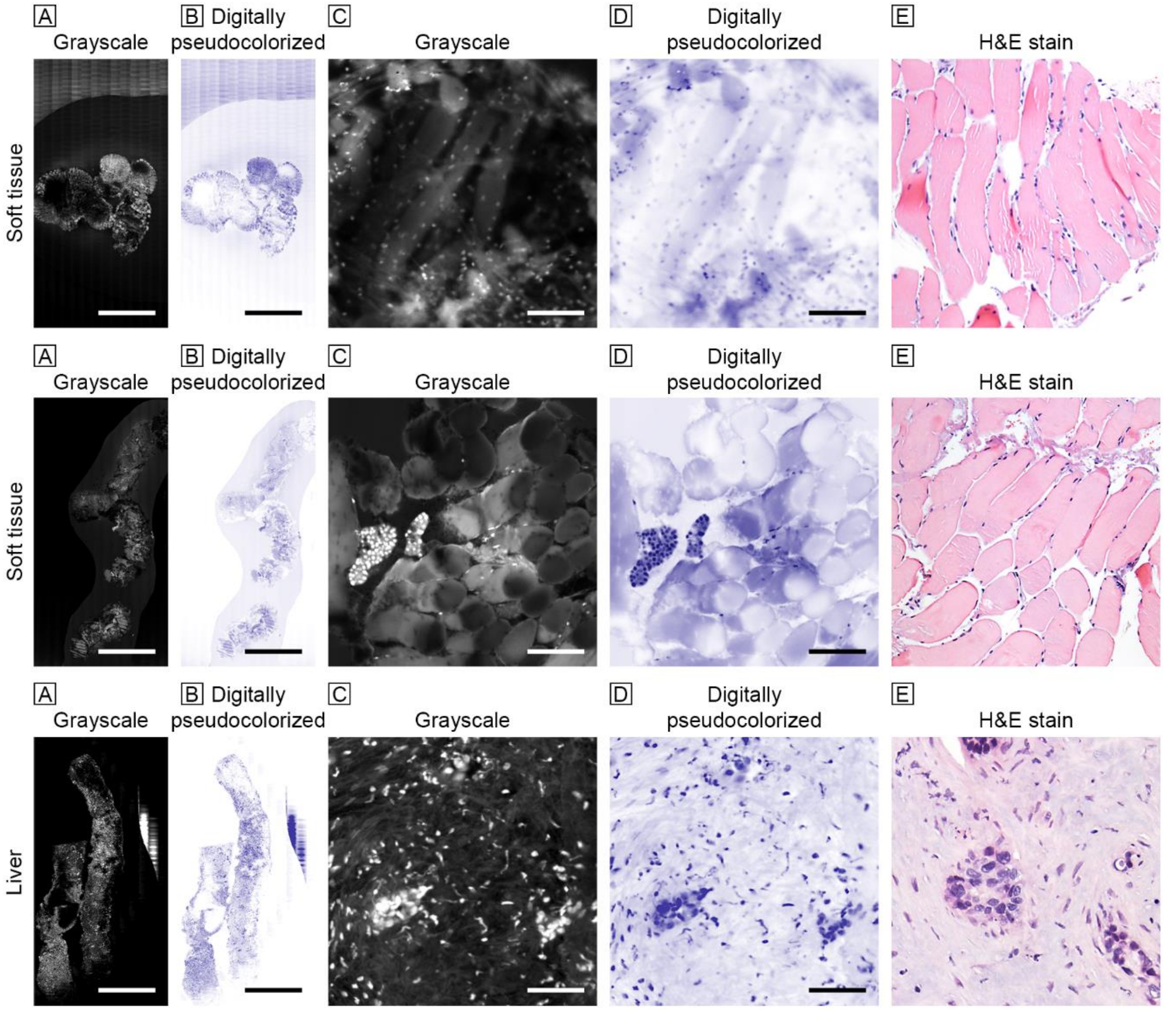

eFigure 2. Specimens From Soft Tissue and Liver

Grayscale (entire core in A, higher magnification in C) and digitally pseudocolorized (entire core in B, higher magnification in D) FCM images and corresponding H&E-stained tissue sections (E) of interventional radiology–guided CNBs including one non-diagnostic CNB from soft tissue (top row) and two suboptimal CNBs from soft tissue and liver (bottom two rows) with less than 20% tumor cellularity. Skeletal muscle alone is seen in the top row that is well recognized in the FCM images. The second row from the bottom shows a soft-tissue biopsy with skeletal muscle and few groups of malignant tumor cells that were noted only on FCM images, not on H&E. The last row shows a liver biopsy with few groups of tumor cells associated with fibrosis, which was well recognized in the FCM images. (Magnification:1500 μM scale bar in columns A and B. 100 μM scale bar in columns C and D. X100 in column E).

©2020 Krishnamurthy S et al. *JAMA Network Open*.
